# Pseudomonas Meningitis Post Spinal Anesthesia: A Case Report

**DOI:** 10.7759/cureus.89501

**Published:** 2025-08-06

**Authors:** Manal El Goubi, Khalid Khaleq, Mohammed Moussaoui

**Affiliations:** 1 Anesthesia and Critical Care, Hôpital Universitaire International Cheikh Khalifa Ibn Zaid, Casablanca, MAR; 2 Anesthesia and Critical Care, Université Hassan II de Casablanca, Casablanca, MAR

**Keywords:** iatrogenic, meningitis, nosocomial, pseudomonas aeruginosa, spinal anesthesia

## Abstract

Nosocomial meningitis following spinal anesthesia is a rare but potentially life-threatening complication that breaches the central nervous system's natural defense barriers. This report presents a case of *Pseudomonas* meningitis post spinal anesthesia, emphasizing the diagnostic, management, and preventive strategies for iatrogenic bacterial meningitis. A 53-year-old patient with sickle cell disease developed febrile confusion 10 days after spinal anesthesia for hemorrhoidal surgery, presenting with meningeal signs and positive infectious markers. Cerebrospinal fluid analysis revealed purulent fluid with leukocytosis, prompting empirical antibiotic therapy. Multi-resistant *Pseudomonas aeruginosa *was identified, necessitating tailored antibiotic treatment and resulting in clinical improvement. Iatrogenic meningitis, though rare, poses significant risks in various medical settings involving spinal procedures. *P. aeruginosa*, identified as the causative agent in this case, presents challenges due to its antibiotic resistance. Early diagnosis, appropriate antibiotic selection, and adherence to hygiene protocols are crucial in managing *P. aeruginosa* infections effectively. Preventive measures, including strict adherence to skin disinfection protocols during invasive procedures, play a vital role in reducing the risk of nosocomial infections and ensuring patient safety post spinal anesthesia.

## Introduction

Nosocomial meningitis after spinal anesthesia is not a common event, but it can be a serious complication with life-threatening repercussions. Because lumbar puncture (for any purpose) breaches all the natural defense barriers of the central nervous system, it clearly serves as a gateway to meningeal infections [1,2]. This can occur through two mechanisms: either due to a lack of rigorous asepsis or through the introduction of bacteria already present in the patient's blood at the time of lumbar puncture, thus creating a meningeal breach [3].

The report presents a case of *Pseudomonas* meningitis following spinal anesthesia. It discusses strategies for diagnosing and managing iatrogenic bacterial meningitis, as well as means of prevention.

## Case presentation

A 53-year-old patient with sickle cell disease developed febrile confusion 10 days after receiving spinal anesthesia for hemorrhoidal surgery at another hospital. Upon admission to Cheikh Khalifa University Hospital, the patient exhibited agitation, a Glasgow score of 12/15, a fever of 40 °C, meningeal stiffness, and a positive Kernig sign. Cerebral MRI results were normal, and laboratory tests indicated a positive infectious work-up. A lumbar puncture revealed purulent cerebrospinal fluid with elevated leukocytes, hyperproteinorachia, and hypoglycorrhachia (Table 1).

**Table 1:** Cerebrospinal fluid analysis results

Parameters	Patient results	Normal values
Appearance	Purulent	Clear
Protein (g/l)	1.16	0.8-0.45
Glucose (mmol/l)	1.9	2.5-3.5
Cell count (cells/mm^3^)	860	< 3
Neutrophils	65%	
Culture	Positive for *Pseudomonas aeruginosa *	

Empirical intravenous (IV) antibiotic therapy was initiated with ceftriaxone (2 g every 12 hours IV), vancomycin (15 mg/kg every 12 hours IV), and gentamicin (5 mg/kg once daily IV). This regimen was maintained for 48 hours. During this period, the patient remained clinically unchanged, with persistent fever (up to 39.2°C), elevated inflammatory markers (C-reactive protein (CRP) > 100 mg/L, WBC count 15,000/mm³), and ongoing neurological symptoms, including lethargy and mild confusion.

Subsequent microbiological culture results identified *Pseudomonas aeruginosa*, classified as multi-drug resistant (MDR) based on non-susceptibility to at least one agent in three or more antimicrobial categories, in accordance with the international consensus definition. Antimicrobial susceptibility testing was performed using the automated VITEK 2 system (bioMérieux SA, Marcy-l'Étoile, France), following European Committee on Antimicrobial Susceptibility Testing (EUCAST) guidelines. The isolate was sensitive to meropenem, amikacin, and ciprofloxacin.

Based on these findings, empirical therapy was discontinued, and targeted antimicrobial therapy was initiated as follows: meropenem 2 g IV every eight hours (administered via extended infusion over three hours), Amikacin 15 mg/kg IV once daily, and ciprofloxacin 400 mg IV every 12 hours. This targeted regimen was continued for a total of 14 days.

Within 72 hours of initiating the targeted antibiotic therapy, the patient exhibited notable clinical improvement, including (i) defervescence: body temperature returned to normal range (< 37.5°C), and remained stable, (ii) neurological recovery: resolution of lethargy and confusion; the patient became alert, oriented, and responsive to verbal commands, and (iii) systemic signs: normalization of heart rate and blood pressure, with improved overall hemodynamic stability. CRP decreased from >100 mg/L to 38 mg/L by day 5 of specific therapy. WBC count declined progressively, reaching 8,500/mm³ by day 7.

The patient regained the ability to sit up, feed orally, and perform basic activities of daily living with minimal assistance by day 10 of therapy. No adverse effects related to meropenem, amikacin, or ciprofloxacin were observed during the treatment course. Renal function remained stable with appropriate monitoring of serum creatinine and amikacin trough levels. The patient was discharged in clinically stable condition upon completion of the 14-day course, with follow-up arranged for continued assessment.

## Discussion

Iatrogenic meningitis is an extremely rare but serious complication that can occur in various settings, including spinal anesthesia, diagnostic lumbar puncture, spinal analgesia, epidural anesthesia, and neurological procedures involving the spinal canal. The exact incidence of infectious complications associated with epidural and spinal anesthesia is unknown. However, several retrospective studies have estimated it to be very low, ranging from 0% to 0.04% [4,5]. The main routes of infection are droplet transmission from the patient or medical staff and needle contamination from inadequately disinfected skin [6]. 

In our case, bacterial meningitis was suspected based on the clinical presentation and the cellularity of the cerebrospinal fluid. *P. aeruginosa* has been identified as the causative agent of the meningitis. While this bacterium is more commonly isolated in nosocomial infections, it is rarely responsible for meningitis. *P. aeruginosa* infections pose challenges due to their inherent or acquired resistance to antibiotics, making them increasingly difficult to manage [7]. 

Traditionally, drugs such as cephalosporins, quinolones, carbapenems, colistin, or aminoglycosides have been used to treat meningitis caused by *P. aeruginosa* [8]. Ciprofloxacin, an antipyocyanic quinolone, is also highly active against it with good meningeal diffusion. However, it must be administered in high doses to achieve the minimum inhibitory concentration, particularly under the condition of confirmed sensitivity on the antibiogram [9]. In recent years, the emergence and spread of MDR strains, especially those resistant to carbapenems, have significantly complicated the treatment of *P. aeruginosa* infections [10]. Our patient received empirical antibiotic treatment with ceftriaxone, gentamicin, and ceftazidime, which was adjusted based on the antibiogram results. The causative organism was sensitive to meropenem, ciprofloxacin, and amikacin.

Early diagnosis of meningitis, whether nosocomial or community-acquired, is crucial to reduce the time between symptom onset and treatment initiation, a critical factor in improving both vital and functional prognosis [1]. Cytological, biochemical, and direct examination results should be promptly available to the medical team managing the patient following a lumbar puncture to ensure appropriate treatment and enhance prognosis. It is essential to rigorously adhere to and systematically follow skin disinfection guidelines during invasive procedures like lumbar punctures, spinal anesthesia, or epidurals to prevent and minimize the risk of nosocomial infections. Thorough cleaning, disinfection, and drying of the puncture site with a sterile compress are essential to prevent such infections. Non-compliance with these guidelines has been linked to a significant number of cases where oral pathogens have been isolated from the cerebrospinal fluid [11,12]. 

Rare cases of *P. aeruginosa* meningitis have been reported following spinal anesthesia, typically associated with nosocomial transmission and lapses in aseptic technique. Successful outcomes have been achieved with early identification and targeted antimicrobial therapy. Additionally, a few reports have identified *Streptococcus salivarius* meningitis linked to contamination during lumbar puncture, where genotypic analysis matched the strain in CSF to that from the clinician’s throat, emphasizing the critical need for strict sterile precautions during neuraxial procedures. [13-15]. Table 2 shows a comparison between some reported cases.

**Table 2:** Comparison of reported cases of meningitis following spinal anesthesia CSF: cerebrospinal fluid; EVD: external ventricular drainage; PFGE: pulsed-field gel electrophoresis

Author	Pathogen	Patient Details (age, sex, country)	Clinical Course	Treatment	Outcome
Andriamamonjisoa et al., 2022 [1]	Pseudomonas aeruginosa	28 years, female, Madagascar	Meningitis developed after spinal anesthesia; diagnosis by CSF culture	Cefoperazone + ciprofloxacin	Full recovery
Kolovani et al., 2020 [16]	Pseudomonas aeruginosa	26 years, female, Albania	Ventricular empyema post-meningitis; EVD placed	Intrathecal colistin + systemic antibiotics	Clinical improvement
Hazim et al., 2019 [17]	Pseudomonas aeruginosa	22 years, female, Saudi Arabia	Meningitis 15 days after spinal anesthesia	Ceftazidime → meropenem + ciprofloxacin	Recovery after 6 weeks
Shewmaker et al., 2010 [15]	Streptococcus salivarius	Two women in labor, United States	Iatrogenic meningitis traced to anesthesiologist’s oropharynx via PFGE	Vancomycin + ceftriaxone	Full recovery

## Conclusions

Bacterial meningitis following spinal anesthesia, although rare, necessitates early diagnosis, pathogen identification, and prompt treatment for optimal outcomes. Prevention relies on strict adherence to hygiene measures across all potential transmission routes and underscores the importance of infection control practices.
